# Supplementary material for: Resolving the full spectrum of human genome variation using Linked-Reads
Source: Genome Res. 2019 Apr;29(4):635–45. doi: 10.1101/gr.234443.118 (PMC6442396; doi:10.1101/gr.234443.118)
Supplement: Supplemental Material [file supp_gr.234443.118_Supplemental_longranger-2.2.zip › longranger-2.2.tar.bz2!/lr2.2-public/lib/stan/cmdstan/stan/doc/api/html/classstan_1_1mcmc_1_1dense__e__metric-members.html]

Stan: Member List


|  |  |
| --- | --- |
|  | Stan  2.5.0  probability, sampling & optimization |


- Main Page
- Related Pages
- Namespaces
- Classes
- Files

- Class List
- Class Index
- Class Hierarchy
- Class Members

All Classes Namespaces Files Functions Variables Typedefs Enumerations Enumerator Friends Macros Pages

- stan
- mcmc
- dense\_e\_metric

stan::mcmc::dense\_e\_metric< M, BaseRNG > Member List

This is the complete list of members for stan::mcmc::dense\_e\_metric< M, BaseRNG >, including all inherited members.

|  |  |  |
| --- | --- | --- |
| base\_hamiltonian(M &m, std::ostream \*e) | stan::mcmc::base\_hamiltonian< M, dense\_e\_point, BaseRNG > | inline |
| dense\_e\_metric(M &m, std::ostream \*e) | stan::mcmc::dense\_e\_metric< M, BaseRNG > | inline |
| dphi\_dq(dense\_e\_point &z) | stan::mcmc::dense\_e\_metric< M, BaseRNG > | inlinevirtual |
| dtau\_dp(dense\_e\_point &z) | stan::mcmc::dense\_e\_metric< M, BaseRNG > | inlinevirtual |
| dtau\_dq(dense\_e\_point &z) | stan::mcmc::dense\_e\_metric< M, BaseRNG > | inlinevirtual |
| err\_stream\_ | stan::mcmc::base\_hamiltonian< M, dense\_e\_point, BaseRNG > | protected |
| H(dense\_e\_point &z) | stan::mcmc::base\_hamiltonian< M, dense\_e\_point, BaseRNG > | inline |
| init(dense\_e\_point &z) | stan::mcmc::base\_hamiltonian< M, dense\_e\_point, BaseRNG > | inlinevirtual |
| model\_ | stan::mcmc::base\_hamiltonian< M, dense\_e\_point, BaseRNG > | protected |
| phi(dense\_e\_point &z) | stan::mcmc::dense\_e\_metric< M, BaseRNG > | inlinevirtual |
| sample\_p(dense\_e\_point &z, BaseRNG &rng) | stan::mcmc::dense\_e\_metric< M, BaseRNG > | inlinevirtual |
| T(dense\_e\_point &z) | stan::mcmc::dense\_e\_metric< M, BaseRNG > | inlinevirtual |
| tau(dense\_e\_point &z) | stan::mcmc::dense\_e\_metric< M, BaseRNG > | inlinevirtual |
| update(dense\_e\_point &z) | stan::mcmc::base\_hamiltonian< M, dense\_e\_point, BaseRNG > | inlinevirtual |
| V(dense\_e\_point &z) | stan::mcmc::base\_hamiltonian< M, dense\_e\_point, BaseRNG > | inline |
| write\_error\_msg\_(std::ostream \*error\_msgs, const std::exception &e) | stan::mcmc::base\_hamiltonian< M, dense\_e\_point, BaseRNG > | inlineprotected |
| ~base\_hamiltonian() | stan::mcmc::base\_hamiltonian< M, dense\_e\_point, BaseRNG > | inline |
| ~dense\_e\_metric() | stan::mcmc::dense\_e\_metric< M, BaseRNG > | inline |


---

[ Stan Home Page ]

*© 2011–2014,
Stan Development Team.*
